# Transportation: Hybrid Splash

**Published:** 2008-06

**Authors:** Harvey Black

Plug-in hybrid electric vehicles (PHEVs) are viewed as a major step in conserving oil and reducing emissions of greenhouse gases and other pollutants. So great is the hope for PHEVs that the U.S. Department of Energy announced in January 2008 it would invest up to $30 million in the development and demonstration of these vehicles, which can run on both gasoline and electricity. A study scheduled for the 15 June 2008 issue of *Environmental Science & Technology* now cautions that the added electric load incurred by charging PHEV batteries would increase the amount of water used by power plants. This isn’t necessarily a deal-breaker, the authors say, but planners need to prepare now to meet the demand.

The big difference between PHEVs and today’s hybrids is the battery. Exisiting hybrids have a smaller battery that’s charged by the gas engine. This is done, for example, by regenerative braking, which converts the energy from the speed of the car (kinetic energy) into electricity to be stored in the battery. A PHEV is similar, but its larger, as-yet undeveloped battery would be charged by plugging it into a household outlet, typically overnight.

The authors analyzed a range of figures on water consumption and withdrawal for both oil refining and electricity generation. Water that is “consumed” ends up in the atmosphere after use, whereas water that is “withdrawn” typically is returned to its source. They found that, compared with gas miles, up to 3 times the water per mile is consumed to power electric miles, and up to 17 times the water is withdrawn. This water is used primarily for cooling at power plants, where electricity is generated by steam-driven turbines. The steam clouds often seen rising from cooling towers indicate waste heat being dissipated into the atmosphere through evaporation.

Study coauthor Michael Webber, a University of Texas engineer who strongly favors the development and use of PHEVs, says local water policy planners need to be aware of this impact. “If they are in a place that has a water-intensive power plant, and they think that plug-in hybrids will be important, they need to be preparing for that future demand because the increased load on the plant can increase the strain on local water resources,” he says. To alleviate this impact, he says, power plants could consider using reclaimed water (e.g., treated wastewater) for cooling.

But Mark Duvall, project manager for electric transportation at EPRI, a nonprofit research organization funded by electric utilities, argues that PHEVs will not measurably increase electric utilities’ water needs. He says utilities are certain to improve the efficiency in the way they use water over the next several decades. “Even if plug-in hybrids become the next big technology and become wildly successful, [the added electricity demand] will still be very small relative to all the electric loads we have today,” he says.

Duvall projects that if PHEVs capture approximately 60% of the auto market by 2050, that will mean an increase in electric capacity of about 4% and a savings of 3–4 million barrels of oil per day. He says the electric sector has been growing by 1–2% annually, but the water withdrawal rate has remained constant.

A crucial question in these calculations is how many PHEVs might eventually take to the roads. Duvall cautions that any estimates are purely speculative at this point and based largely on focus groups and similar tools. According to a survey cited in the 2001 EPRI report *Comparing the Benefits and Impacts of Hybrid Electric Vehicle Options*, more than half of respondents would pay 26.5% more for a hybrid than a conventional car if the hybrid could go 60 miles per charge cycle powered solely by electricity. Just over 7% would pay nearly 80% more for such a car.

But Duvall notes that actual customer behavior may be altered by a variety of conditions that were not present when the survey was done, such as gas prices. He says about 1 million regular hybrids are on the road right now, and he expects there may be a similar number between 2010 and 2018. Meanwhile, Saturn and Toyota in early 2008 announced they plan to market PHEVs within the next two years.

